# Hydrogel Phase Transition‐Driven Soft Gating Circumvents Debye Screening for Advanced Biosensing

**DOI:** 10.1002/advs.78049

**Published:** 2026-09-28

**Authors:** Liu Shi, Zimeng Zhang, Bingheng Li, Yalei Gao, Ruirui Zhang, Zheying Mu, Jian Ni, Bing Bo, Genxi Li

**Affiliations:** ^1^ School of Environmental and Biological Engineering Nanjing University of Science and Technology Nanjing People's Republic of China; ^2^ State Key Laboratory of Analytical Chemistry For Life Science School of Life Sciences Nanjing University Nanjing People's Republic of China; ^3^ Department of Medical Oncology Shanghai Pulmonary Hospital & Thoracic Cancer Institute Shanghai Pulmonary Hospital Tongji University School of Medicine Shanghai People's Republic of China; ^4^ Center For Molecular Recognition and Biosensing School of Life Sciences Shanghai University Shanghai People's Republic of China

**Keywords:** advanced biosensing, biomimetic gating, Debye screening effect, hydrogel, phase transition

## Abstract

Solid‐state nanofluidic sensors provide a versatile platform for label‐free molecular diagnostics. However, achieving high sensitivity in complex physiological environments remains severely restricted by the Debye screening effect. Here, we introduce a stimuli‐responsive 3D soft gating strategy based on a hydrogel phase transition to address this limitation. By asymmetrically assembling a peptide‐DNA hybrid hydrogel on the outer surface of nanochannels, we establish a volumetric functional zone governed by macroscopic Donnan equilibrium. Target‐induced structural disassembly of this network triggers a synergistic gating response, simultaneously abolishing Donnan enrichment and decreasing interfacial wettability. This dual‐parameter modulation significantly alters transmembrane ion flow, generating a substantial shift in ionic current. By shifting the dominant physics from 2D surface electrostatics to this 3D volumetric Donnan effect, the platform effectively circumvents Debye screening in high‐ionic‐strength media. Targeting the immune effector Granzyme B (GrzB), the soft gating sensor (SGS) achieves an ultralow detection limit of 0.830 fM. Clinically, the SGS has successfully tracked the longitudinal dynamics of serum GrzB in lung cancer patients undergoing immunotherapy. The high diagnostic accuracy confirms the capability of this SGS to operate directly in unpurified physiological fluids, providing a robust analytical tool for advanced molecular diagnostics.

## Graphical abstract

A 3D soft gate utilizes a macroscopic hydrogel phase transition to effectively circumvent Debye screening in high‐salt environments. The intact matrix maximizes transmembrane ion flux via volumetric Donnan enrichment and superhydrophilicity. Target‐triggered network disassembly abolishes these effects, introducing a synergistic physical barrier. This macroscopic dual‐gating mechanism enables direct, ultrasensitive biomarker detection in unpurified clinical serum.

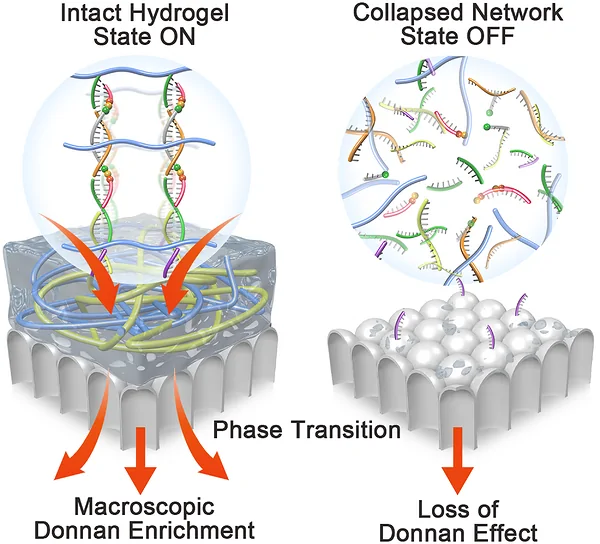

## 1 Introduction

Biological ion channels operate as sophisticated soft molecular machines that orchestrate transmembrane ion transport through dynamic gating mechanisms. Instead of relying solely on rigid steric exclusion [1], they achieve high selectivity and efficiency by coupling the local electrostatic environment with the interfacial hydration status in response to external stimuli [2, 3]. For instance, potassium channels utilize synergistic amino acid residues to strip the hydration shell of ions via electrostatic coordination [4, 5], achieving transport rates approaching the diffusion limit [6, 7]. Inspired by these biological soft machines, biomimetic ionic gates based on solid‐state nanochannels have emerged as robust platforms in iontronics and biosensing [8, 9, 10, 11]. To emulate biological gating within mechanically stable synthetic nanochannels, researchers functionalized the rigid surfaces with stimuli‐responsive molecular elements [12, 13, 14]. This strategy enables the direct transduction of molecular recognition into measurable ionic signals [15, 16]. Recent elegant designs have utilized diverse probes to achieve this, including charged peptides for tumor biomarker detection [17], specific cyclic peptides for distinguishing post‐translational modifications [18], polymer brushes to mimic neurochemical signal transduction [19], and diblock DNA for elucidating charge regulation [20]. Ultimately, this highly sensitive, label‐free approach delivers reliable quantitative performance, demonstrating immense potential in fields such as next‐generation gene sequencing, clinical diagnostics, and environmental monitoring.

Classical solid‐state nanofluidic sensors achieve superior sensitivity in ideal, low‐ionic‐strength buffers by utilizing the overlap of the electric double layer (EDL) to electrostatically regulate ion flux [21, 22, 23]. However, translating these platforms to complex physiological environments, such as whole blood, serum, or urine [24, 25], is fundamentally hindered by the Debye screening effect [26, 27]. In these high‐ionic‐strength matrices, abundant background electrolytes severely compress the EDL, reducing the Debye length to sub‐nanometer scales [27, 28, 29, 30]. This extreme compression completely neutralizes the electrostatic field generated by conventional surface probes, preventing them from modulating ion flow at the channel center [31]. Consequently, this inherent screening effect abolishes sensor sensitivity, constituting a major hurdle for clinical immunodiagnostics [32]. Attempts to bypass this charge screening using pure steric hindrance strategies have proven inadequate, suffering from sluggish response kinetics and limited signal modulation when targeting trace biomarkers. Therefore, developing a gating strategy that effectively circumvents the Debye screening limit through a shift from 2D surface electrostatics to 3D volumetric regulation, synergistically coupling interfacial wettability, remains a critical imperative.

To effectively circumvent this thermodynamic limitation, we introduce a biomimetic external soft gating strategy that shifts the fundamental design principle from conventional 2D surface functionalization to 3D volumetric regulation. By asymmetrically assembling a highly versatile peptide‐DNA hybrid hydrogel on the exterior of anodic aluminum oxide (AAO) nanochannels, we create a volumetric functional zone that functions as an active ion trap. Its superhydrophilic and highly charged network pre‐concentrates bulk ions via macroscopic Donnan enrichment to minimize geometric access resistance and simultaneously generates a substantial Donnan potential, collectively resisting ionic screening effects in physiological environments. To demonstrate the advanced biosensing utility and superior analytical performance of this platform, we have applied it to the ultrasensitive profiling of Granzyme B (GrzB), a pivotal biomarker for evaluating lung cancer immunotherapy efficacy [33, 34]. As illustrated in Scheme 1a, the intact 3D hydrogel network acts as an ion trap (State ON), maintaining a high space charge density and superhydrophilicity at the channel entrance to maximize ion flux (Scheme 1b) [35]. In the presence of GrzB, enzymatic cleavage of the peptide cross‐linkers triggers the disassembly of the hydrogel network. This macroscopic phase transition induces a synergistic shutdown effect wherein the abolishment of Donnan enrichment, which dissipates the localized charge reservoir, is coupled with a decay in interfacial wettability from superhydrophilic to hydrophilic, as depicted in Scheme 1c. Consequently, the energy barrier for ion entry is drastically elevated, leading to a marked reduction in ionic current (State OFF) (Scheme 1d). By exploiting the inherent modularity of the building blocks, we have further demonstrated the capacity of the platform for advanced biological computing via multiplexed logic operations. Ultimately, by effectively suppressing complex serum background interference, this 3D soft gate enables precise longitudinal assessment of clinical lung cancer immunotherapy. This successful clinical validation demonstrates its robust capability to operate directly in complex physiological matrices, establishing a reliable and practical platform for advanced immunodiagnostics.

**SCHEME 1:**
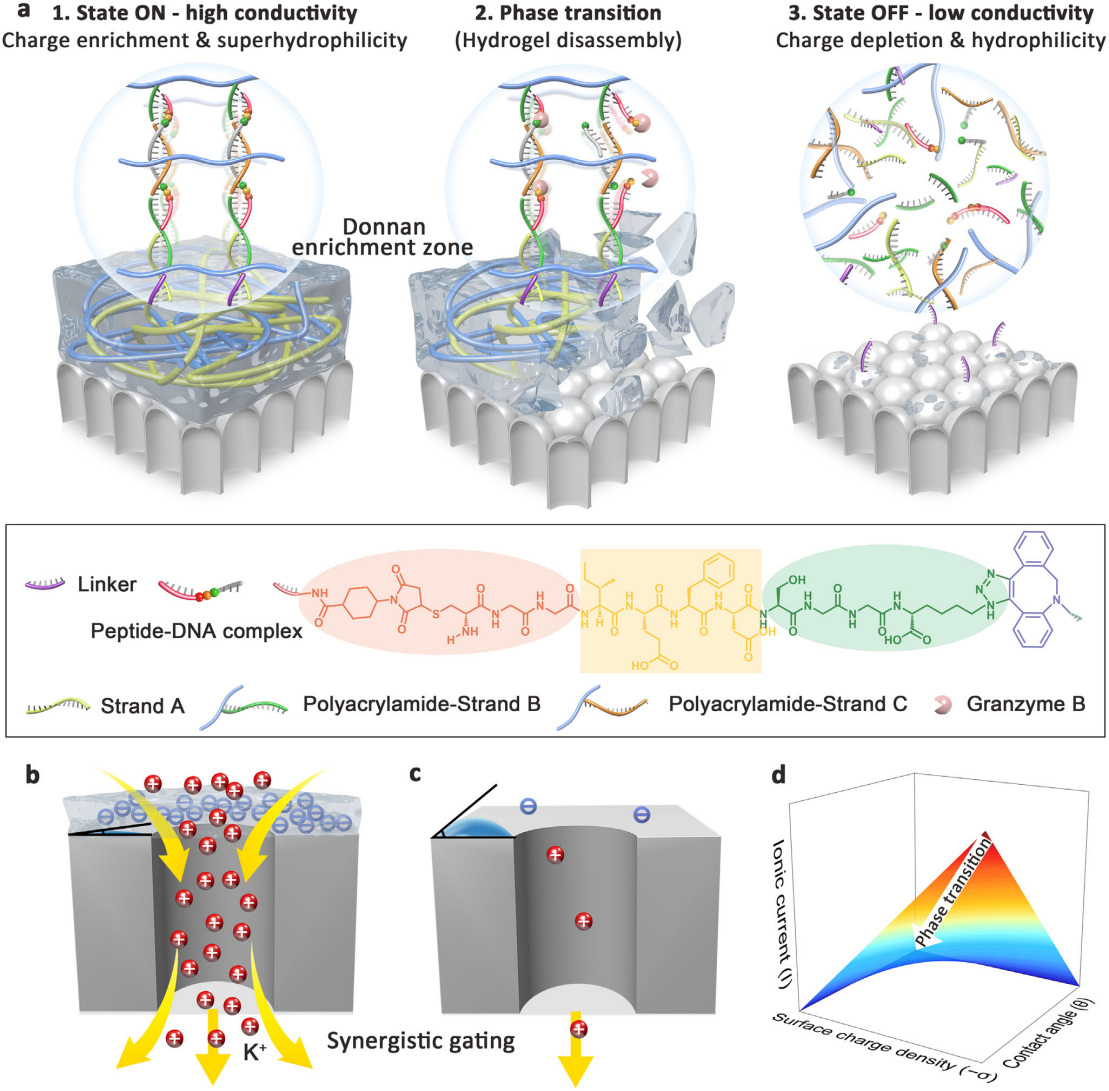
Design and working principle of the hydrogel‐phase‐transition‐driven external soft gating system. (a) Schematic illustration of the biomimetic sensing mechanism based on target‐triggered hydrogel disassembly. (b,c) Cross‐sectional views illustrating ion transport dynamics in the intact hydrogel state (b) and following GrzB‐triggered disassembly (c). (d) Theoretical energy landscape mapping the ionic current response against surface charge density and contact angle.

## 2 Results and Discussion

### 2.1 Construction and Characterization of the Soft Gating Interface

As the physical foundation for the 3D volumetric regulation strategy, a stimuli‐responsive peptide‐DNA hybrid hydrogel was asymmetrically assembled on the outer surface of an anodic aluminum oxide (AAO) membrane (Scheme 1a and Table S1) [36, 37, 38]. The hierarchical assembly of this supramolecular network is driven by a localized proximity effect. The molecular‐level details of this sequential hybridization on the channel surface, serving as the building blocks for the 3D network, are schematically illustrated in Figure S1. Specifically, single‐stranded linker DNA was first covalently immobilized onto the nanochannel surface to serve as the structural anchor. A bridging strand (SA) was then introduced to hybridize with both the linker DNA and polyacrylamide‐strand B (P‐SB) conjugate. Subsequently, a peptide‐DNA complex (PDC) cross‐linker was integrated as the core recognition element to simultaneously hybridize with P‐SB and polyacrylamide‐strand C (P‐SC) conjugate. The intact peptide backbone of the PDC physically tethers these polymer‐nucleic acid architectures in close spatial proximity. This localized proximity effect entropically stabilizes the short DNA duplexes, driving the robust cross‐linking of the 3D hydrogel network. Conversely, specific enzymatic cleavage of the PDC by GrzB severs this physical linkage, instantly abolishing the proximity effect. This loss drastically destabilizes the thermodynamic equilibrium, triggering the spontaneous dehybridization of the short duplexes and the macroscopic collapse of the soft gating interface.

To construct this sophisticated network, the full PDC cross‐linker was synthesized from precursor peptide 1 (Table S2 and Figure S2) via a stepwise orthogonal strategy involving thiol‐maleimide coupling and strain‐promoted alkyne‐azide cycloaddition (SPAAC) (Figure 1a and Figure S3) [39, 40, 41]. Subsequently, polyacrylamide gel electrophoresis (PAGE) validated the stepwise molecular weight shifts during the PDC conjugation, as well as the complete disappearance of the PDC band following specific enzymatic cleavage by GrzB (Figure 1b) [42, 43]. Prior to chemical modification, the physical morphology of the bare AAO substrate was characterized. Atomic force microscopy (AFM) revealed a uniform, high‐density nanochannel array on the membrane surface (Figure 1c,d). Furthermore, scanning electron microscopy (SEM) confirmed the asymmetric structure of the AAO membrane, clearly displaying a distinct large‐channel base (∼60 nm in diameter) and a limiting‐pore surface (0–1 nm). The cross‐sectional view revealed a total membrane thickness of ∼60 µm and highlighted the presence of the intact barrier layer (Figure S4).

**FIGURE 1:**
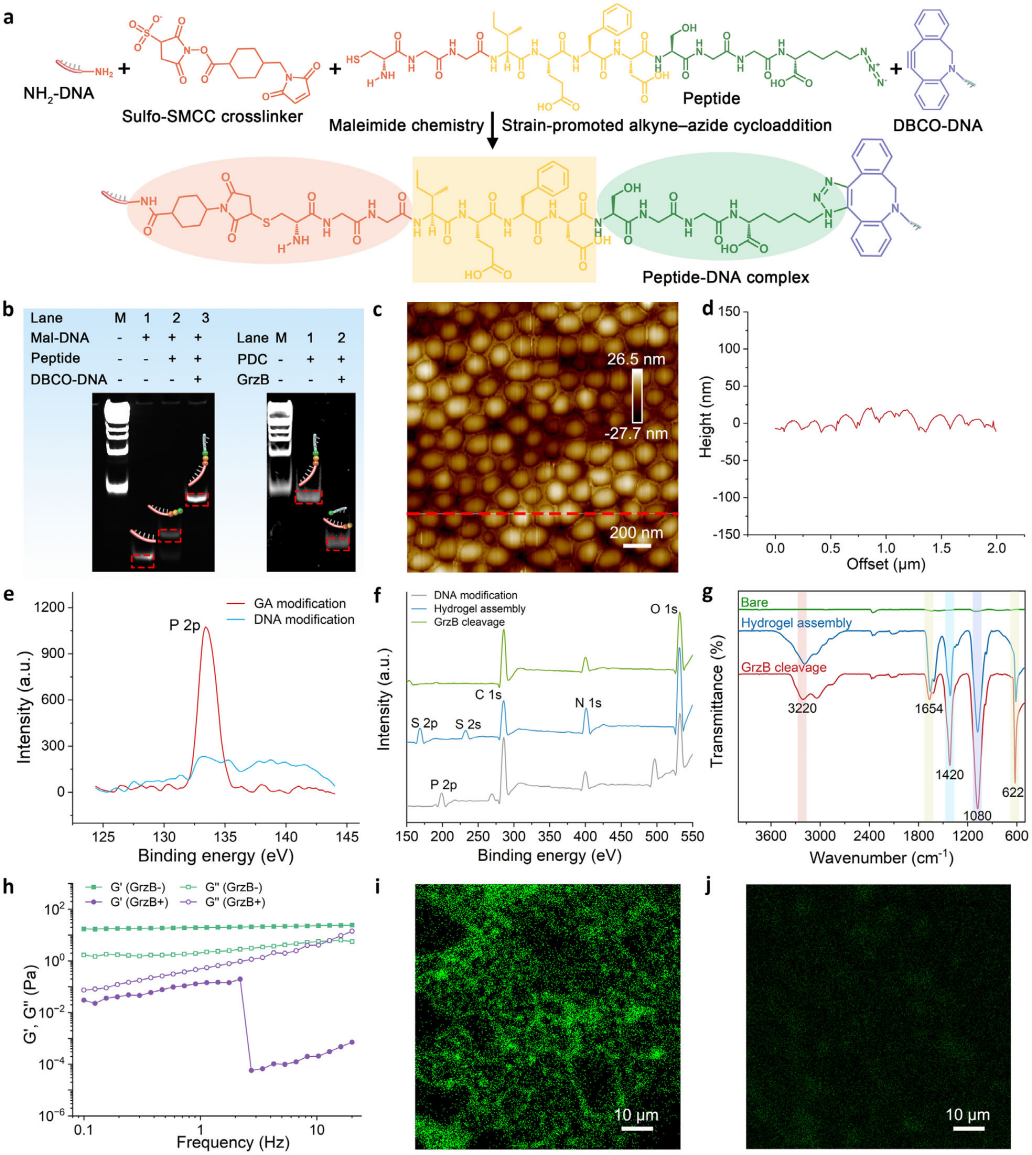
Synthesis and characterization of the functional peptide‐DNA hydrogel architecture. (a) Synthetic route of the Peptide‐DNA Complex (PDC) cross‐linker via orthogonal thiol‐maleimide coupling and strain‐promoted alkyne‐azide cycloaddition (SPAAC). (b) Polyacrylamide gel electrophoresis (PAGE) analysis of the stepwise conjugation and enzymatic cleavage of the PDC. (c,d) AFM characterization of the bare AAO nanochannel array. (e,f) XPS high‐resolution spectra (e) and wide‐scan survey (f) monitoring elemental evolution across DNA modification, in situ hydrogel assembly, and GrzB‐triggered disassembly. (g) FTIR spectra confirming the surface chemical changes before and after GrzB digestion. (h) Rheological frequency sweeps of the hydrogel before (GrzB‐, green) and after (GrzB+, purple) target‐induced cleavage. (i,j) Confocal laser scanning microscopy (CLSM) images visually validating the macroscopic phase transition of the fluorescently labeled hydrogel network upon cleavage.

The hydrogel‐gated solid‐state nanochannels were constructed via a sequential chemical functionalization (Figure S5), involving initial (3‐aminopropyl)triethoxysilane (APTES) aminosilanization of the bare AAO surface, glutaraldehyde (GA) activation, and subsequent covalent immobilization of the single‐stranded linker DNA. High‐resolution X‐ray photoelectron spectroscopy (XPS) systematically verified the sequential chemical functionalization steps. The Si 2p spectrum confirmed APTES silanization on the bare AAO (Figure S6), while subsequent DNA immobilization was evidenced by a strong P 2p signal (Figure 1e). Furthermore, the emergence of amplified N 1s and distinct S 2p peaks confirmed the integration of sulfur‐containing peptide cross‐linkers, validating the in situ hydrogel assembly (Figure 1f and Figure S7). Conversely, their marked attenuation following GrzB cleavage quantitatively verified the hydrogel disassembly.

Fourier‐transform infrared (FTIR) spectroscopy provided complementary molecular evidence for the dynamic transition. Hydrogel assembly yielded a characteristic amide I band (1654 cm^−^^1^) and a broad hydrogen‐bonding network peak (3220 cm^−^^1^) [44], while bands at 1080 and 1420 cm^−^^1^ confirmed the DNA‐peptide immobilization. Crucially, GrzB cleavage caused a decrease at 1654 and 3220 cm^−^^1^, reflecting targeted peptide digestion. The spontaneous dehybridization left only the covalently anchored linker DNA, as corroborated by the persistence of the 1080 and 1420 cm^−^^1^ signals (Figure 1g). Macroscopically, vial inversion tests demonstrated the phase transition of the hydrogel from an intact 3D network to a flowing state upon GrzB incubation (Figure S8). To quantitatively verify this macroscopic target‐induced phase transition, rheological measurements were performed (Figure 1h). In the absence of GrzB, the storage modulus (*G′*) was higher than the loss modulus (*G′′*), confirming the formation of a stable 3D crosslinked network. Following GrzB incubation, however, the rheological behavior completely inverted (*G′′* >*G′*). The collapse of *G′* indicates the total loss of the elastic network, providing physical evidence for the gel‐to‐sol transition [45]. Microscopically, confocal laser scanning microscopy (CLSM) provided direct visual evidence that the intense fluorescence of the intact 5‐carboxyfluorescein (FAM)‐labeled hydrogel (Figure 1i) significantly diminished after enzymatic treatment (Figure 1j), confirming the successful disassembly of the network on the outer surface of the nanochannels.

### 2.2 Phase Transition‐Driven Synergistic Effect on Ion Transport

The fundamental mechanism driving signal amplification in this system is the phase collapse of the hydrogel, which induces a synergistic dual‐shutdown effect. First, cross‐sectional scanning electron microscopy (SEM) visualized the interfacial architecture. In the initial state, a thick, porous 3D supramolecular hydrogel network densely covered the AAO nanochannels (Figure 2a and Figure S9). Following GrzB‐triggered disassembly, this external network was efficiently removed, re‐exposing the underlying substrate (Figure 2b). This structural phase transition was further corroborated by atomic force microscopy (AFM) topographical analysis. The intact hydrogel presented a distinctly roughened surface morphology (Figure 2c) with an elevated height profile (Figure 2e). Conversely, targeted enzymatic cleavage induced a macroscopic network collapse (Figure 2d), leading to a reduction in overall surface roughness and height (Figure 2f). As illustrated in the schematics (Figure 2g,h), this structural degradation considerably altered the interfacial physical chemistry. Water contact angle (CA) measurements validated the progressive modulation of interfacial wettability (Figure 2i and Figure S10). While the initial APTES modification yielded a moderately hydrophobic surface (∼68.4°), subsequent hydrogel assembly induced a substantial transition to a superhydrophilic state (∼7.6°). Crucially, targeted enzymatic cleavage caused a decay in wettability, with the CA reverting to a less hydrophilic state (∼39.0°). Concurrently, ζ potential and surface charge density calculations demonstrated the dissipation of localized negative charges (Figure 2j,k). Specifically, the intact 3D hydrogel acted as a dense volumetric charge reservoir, yielding a highly negative effective surface charge density of −5.26 mC/m^2^. Upon targeted cleavage, this localized charge density sharply decreased to −0.92 mC/m^2^, representing an ∼82.5% loss in charge capacity. This simultaneous dissipation of surface charge and decay in wettability shifted the system down a theoretical energy landscape (Scheme 1d), synergistically elevating the energy barrier for ion entry [46]. Within this dual‐gating mechanism, macroscopic Donnan enrichment plays the primary role by securing continuous ion flux in high‐salt environments [47]. Interfacial wettability acts as a secondary amplifier. Upon target cleavage, the hydrogel network collapses, and the decreased wettability introduces a physical barrier that further restricts ion transport.

**FIGURE 2:**
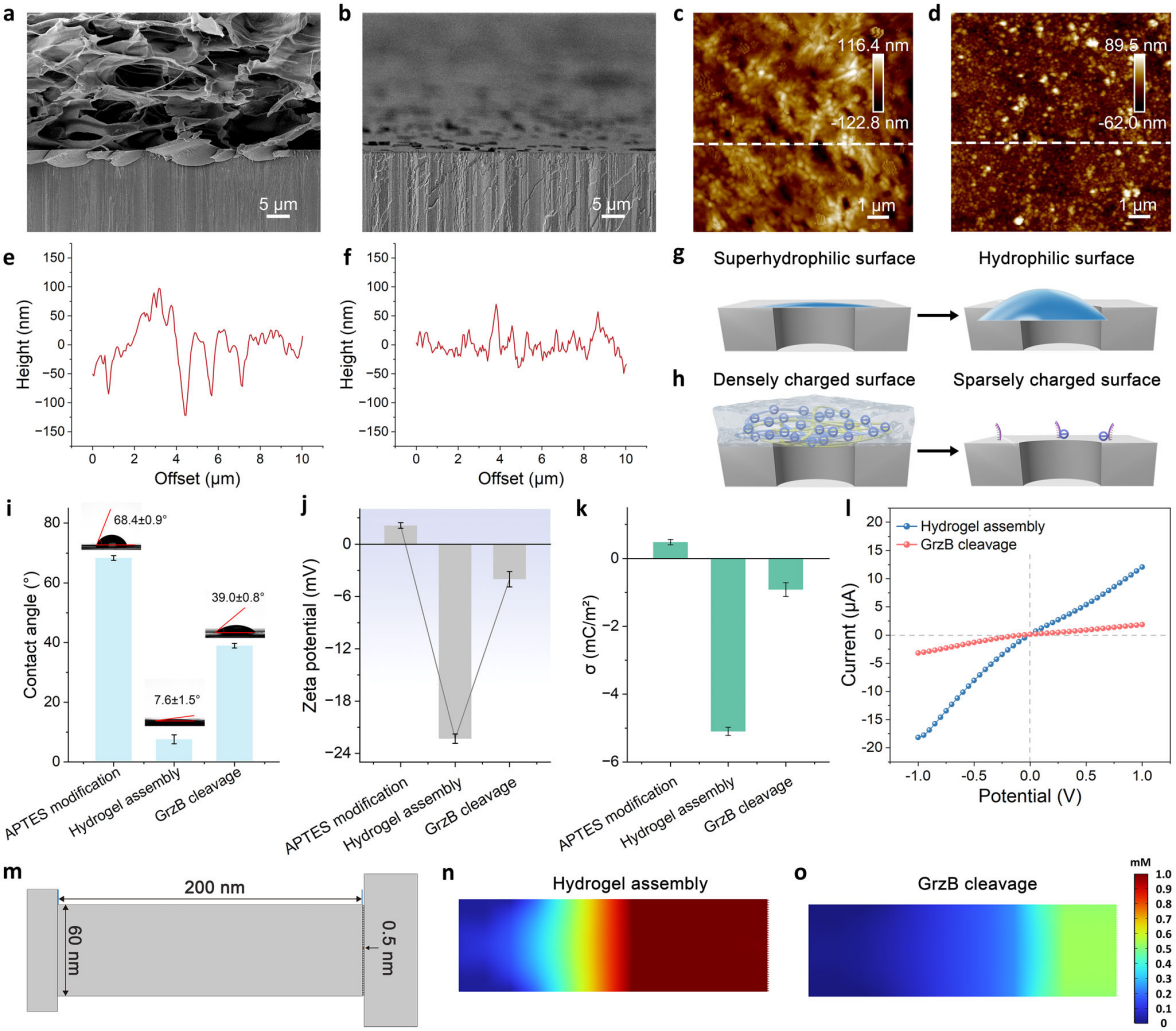
Characterization of the hydrogel phase transition and its synergistic regulation of ion transport. (a–f) Cross‐sectional SEM (a,b) and AFM (c–f) images revealing the structural morphology of the gating interface in the intact hydrogel state (a,c,e) and the post‐cleavage state (b,d,f). (g,h) Schematics illustrating the simultaneous phase‐transition‐induced changes in interfacial wettability and surface charge. (i–k) Verification of synergistic dual‐parameter modulation before and after enzymatic degradation, including water contact angle decay (i), zeta potential shifts (j), and the calculated dissipation of effective surface charge density (k). (l) Current–voltage (*I–V*) characteristics demonstrating the sharp reduction in ionic conductance from the ON to OFF state. (m–o) Finite‐element simulations based on the 2D model (m) showing the transmembrane cation concentration distributions for the intact (n) and disassembled networks (o). Data in (i–k) are mean ± SD (n = 3).

To quantitatively evaluate these transmembrane ionic transport properties, a two‐compartment H‐type electrochemical cell was employed, with the functionalized AAO membrane mounted between two Ag/AgCl electrodes (Figure S11). The device exhibited a sharp reduction in ionic conductance, transitioning from a high‐current ON state (∼17.8 µA at −1.0 V) to a low‐current OFF state (∼3.07 µA) upon GrzB treatment, yielding a highly competitive ON/OFF gating ratio of ∼5.8 (Scheme 1b,c and Figure 2l). To theoretically elucidate these dynamics, finite‐element simulations governed by the Poisson–Nernst–Planck equations were conducted, utilizing these experimentally derived charge densities and wetting states as precise boundary conditions (Figure 2m) [48]. The steady‐state cation concentration maps confirmed that the highly charged, hydrophilic intact hydrogel acted as an ion trap, inducing significant cation enrichment via the Donnan effect to maximize transmembrane flux (Figure 2n). Upon phase disassembly, the concurrent dissipation of the volumetric charge reservoir and decay in wettability completely abolished this enrichment zone and elevated the transport resistance (Figure 2o), corroborating the experimental current–voltage (*I–V*) responses. While the current macroscopic Donnan model is robustly supported by these DC measurements and theoretical simulations, macroscopic ion transport is inherently multifaceted. Future studies incorporating complementary independent parameters, such as permselectivity, will further refine this physical framework.

### 2.3 Amplified Biosensing and the Necessity of the 3D Volumetric Zone

Systematic optimization of key interfacial parameters, including nanochannel diameter (Figure S12), linker DNA concentration (Figure S13), hydrogel network density and molar ratio (Figures S14 and S15), and reaction kinetics (Figures S16–S20), was conducted to maximize the signal gating ratio for biosensing. Under optimal conditions, the hydrogel‐gated device exhibited great analytical performance. The ionic current decreased monotonically with increasing GrzB concentrations from 1 fM to 10^6^ fM (Figure 3a,b). The calibration curve revealed a highly linear relationship between the current blockade ratio (*y*) and the logarithm of the target concentration (*x*), yielding a linear regression equation of *y* = 0.137 + 0.121 lg *x* (*R* ^2^ = 0.988) (Figure 3c).

**FIGURE 3:**
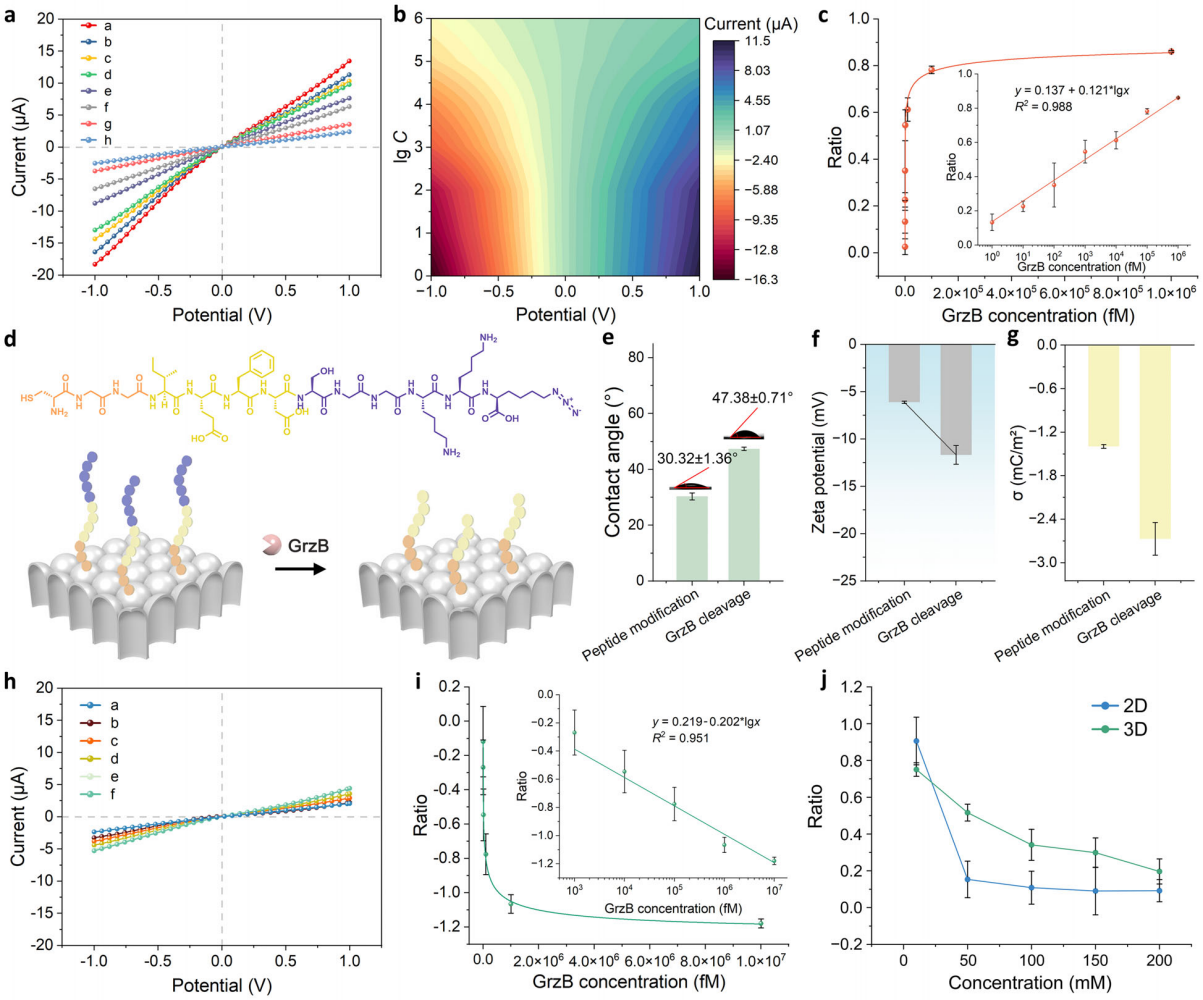
Ultrasensitive detection performance and mechanistic comparison with a 2D monolayer control. (a,b) *I*–*V* characteristics (a) and 2D contour map (b) of the 3D hydrogel‐gated device upon exposure to GrzB at concentrations ranging from 1 to 10^6^ fM (curves a–h). (c) Calibration curve plotting the current blockade ratio against GrzB concentration. The inset demonstrates a robust linear correlation between the response ratio and the logarithmic target concentration (lg *C*). (d) Schematic of the traditional 2D peptide monolayer control engineered on the AAO surface. (e–g) Characterization of the 2D interface properties: contact angle (e), zeta potential (f), and calculated surface charge density (g) before and after GrzB cleavage. (h,i) Corresponding *I–V* response (h) and calibration curve (i) of the 2D control device for detecting GrzB. (j) Comparison of the gating ratios between the 2D surface‐modified nanochannels and the 3D peptide‐DNA hydrogel‐gated nanochannels as a function of background ionic strength (10–200 mM). Data in (c,e,f,g,i,j) are mean ± SD (n = 3).

The fundamental role of the 3D phase transition in achieving this ultrasensitivity was verified by evaluating a traditional 2D monolayer control, engineered using a tailored precursor, Peptide 2 (Figure 3d and Figure S21 and Table S2). In contrast to the significant physical shifts observed in the 3D system, the 2D interface exhibited only relatively minor variations in wettability and surface charge density after enzymatic cleavage (Figure 3e–g and Figure S22) [49]. Consequently, the 2D control suffered from a highly constrained dynamic range and substantially reduced sensitivity (*R* ^2^ = 0.951) (Figure 3h,i and Figure S23). Ultimately, by effectively circumventing the Debye screening limitations through the volumetric charge‐wettability synergy, our 3D hydrogel gating strategy achieved a notably low limit of detection (LOD) of 0.830 fM (3σ/slope), outperforming the 2D control and other reported sensing platforms (Figure S24 and Table S3) [50, 51, 52, 53, 54, 55, 56].

To investigate the effect of Debye screening on the system, we evaluated the gating ratio as a function of background ionic strength (10–200 mM). As shown in Figure S25, the 2D surface‐modified nanochannels exhibited a sharp decline in the gating ratio at ∼50 mM. At such high ionic strengths, the severely compressed Debye length (*λ*_D_ ≈ 0.8 nm at 150 mM) restricts the electrostatic field to the immediate solid wall, leaving the bulk channel ungated [57]. In comparison, the 3D platform maintained robust signal modulation up to 200 mM (Figure S26 and Figure 3j). This robustness stems from a critical difference in physical length scales, in that the macroscopic thickness of the 3D hydrogel (∼25–30 µm) is orders of magnitude larger than both its internal mesh size (∼60 nm, estimated from the rheological modulus) and the compressed *λ*_D_ [58]. Consequently, rather than relying on localized surface electrostatics, this massive 3D matrix operates under bulk Donnan equilibrium. By trapping counter‐ions via macroscopic volumetric Donnan enrichment, the micrometer‐thick hydrogel network effectively mitigates the Debye screening effect typically observed in planar architectures. To explicitly evaluate the quantitative performance under physiological conditions, target calibration was performed in a 150 mM buffer (Figure S27) to enable a systematic comparison with the baseline parameters at 10 mM. While the platform demonstrated a maximized gating ratio, an ultralow LOD of 0.830 fM, and a high calibration *R* ^2^ of 0.988 at 10 mM, the sensing performance understandably attenuated at 150 mM, yielding a reduced gating ratio (Figure 3j), an LOD of 133.60 fM, and an *R* ^2^ of 0.967. Despite this expected attenuation, the fact that reliable quantitative signal modulation is successfully maintained at 150 mM confirms that the 3D strategy effectively buffers against the Debye screening effect, highlighting its robust potential for target detection in physiological environments.

### 2.4 Transition to Biocomputing and Device Robustness

The inherent modularity of the supramolecular network enables the translation of multiplexed biochemical recognition events into advanced biocomputing operations. To achieve this, GrzB‐specific peptide 1 and matrix metalloproteinase 2 (MMP‐2)‐specific peptide 3 (Table S2 and Figures S28 and S29) were simultaneously integrated into a unified hydrogel network, endowing the platform with multi‐input parallel processing capabilities (Figure 4a). The distinct current blockade ratios generated by various input combinations were comprehensively mapped via a multidimensional radar plot (Figure 4b). Based on these programmable responses, we conceptualized and constructed a cascaded biological logic circuit operating via two parallel INHIBIT sub‐circuits. The presence of either target enzyme initiates the cleavage cascade, while their respective specific inhibitors effectively veto this process. Because either uninhibited enzymatic pathway can independently induce phase disassembly to generate a measurable current response, the integrated system operates as an OR gate. Mathematically, the final digital output (*Out*) of this intelligent sensor can be rigorously described by the following Boolean logic equation:

$$OUT=\left(w\wedge \neg x\right)\vee \left(y\wedge \neg z\right)$$

where ∧ represents the logical AND, ¬ denotes the logical NOT, and ∨ signifies the logical OR. The device executed the predefined multidimensional truth table (Figure 4c,d). This sophisticated logic gating mechanism, supported by molecular docking simulations of the precise structural binding between the enzymes and their inhibitors (Figure 4e), demonstrates the versatility of the dual‐target platform and showcases its potential for highly accurate disease screening in complex physiological environments.

**FIGURE 4:**
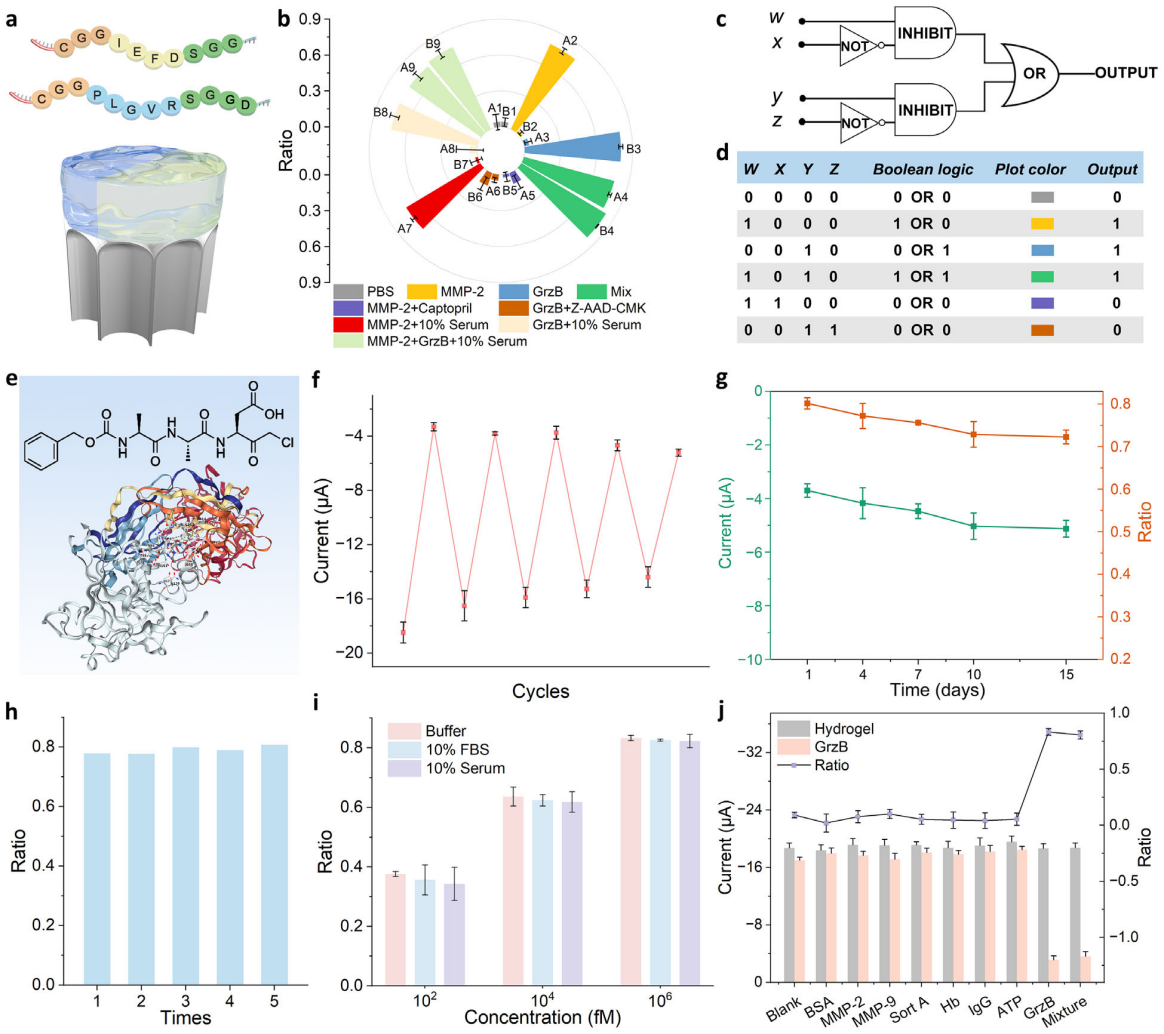
Construction of a biological biocomputing logic gate and evaluation of device robustness. (a–d) Design and execution of a cascaded INHIBIT‐OR logic gate system. The dual‐functionalized hydrogel network (a) processes four distinct biochemical inputs: GrzB (w), its inhibitor Z‐AAD‐CMK (x), matrix metalloproteinase 2 (MMP‐2) (y), and its inhibitor Captopril (z). The resulting current blockade ratios are mapped in a multidimensional radar plot (b), with the equivalent logic circuit (c) and truth table (d) confirming the successful INHIBIT‐OR operations. (e) Molecular docking simulation elucidating the binding interaction between GrzB and Z‐AAD‐CMK. (f–h) Evaluation of device robustness and physical stability, demonstrating consistent current switching over 5 regeneration cycles (f), long‐term stability over a 15‐day storage period (g), and excellent reproducibility across five independent batches (h). (i,j) Assessment of anti‐interference capability and selectivity, verifying reliable detection in complex biological matrices (10% fetal bovine serum (FBS) and 10% human serum) (i) and specific profiling against a panel of potential interferents (j). All measurements were performed in triplicate. Data in (b,f,g,i,j) are mean ± SD (n = 3).

To ensure reliable operation in complex biological environments, we systematically evaluated the physical robustness and anti‐fouling capabilities of the phase‐transition interface. The system exhibited highly consistent current switching over 5 regeneration cycles (Figure 4f), supported by the fully reversible wettability tracked during the re‐cleavage process (Figures S30 and S31). Negligible signal degradation was observed over a 15‐day storage period (Figure 4g), alongside great batch‐to‐batch reproducibility (RSD = 1.62%, *n* = 5) (Figure 4h). Furthermore, the intact hydrogel functioned effectively as a physical shield against non‐specific fouling [59, 60]. The sensor maintained its high detection performance in complex matrices such as 10% fetal bovine serum (FBS) and 10% human serum (Figure 4i), and exhibited strict selectivity against a panel of potential protein interferents (Figure 4j).

### 2.5 Clinical Application for Immunotherapy Monitoring

During effective immune checkpoint blockade (ICB) therapy, activated cytotoxic T lymphocytes release substantial amounts of GrzB to induce targeted tumor cell apoptosis (Figure 5a) [61]. Consequently, the dynamic tracking of serum GrzB serves as a powerful indicator of therapeutic response. To evaluate our platform in a clinical context, serum samples were collected from healthy donors (n = 5) and lung cancer patients both before (n = 10) and after (n = 10) ICB treatment. The study protocol was approved by the Medical Ethics Committee of Shanghai Pulmonary Hospital (Approval No. K22‐047Z) and was conducted in strict accordance with the ethical principles of the Declaration of Helsinki, with written informed consent obtained from all participants. Leveraging the inherent anti‐fouling robustness of its 3D supramolecular network, the soft gating sensor (SGS) was employed to directly analyze these unpurified clinical specimens (Figure 5b). The quantitative results revealed distinct clinical profiles (Figure 5c). Specifically, healthy individuals exhibited baseline GrzB concentrations averaging ∼0.8 pM, whereas pre‐treatment patients showed significantly diminished levels (∼0.3–0.5 pM), reflecting the immunosuppressive microenvironment of advanced malignancies. Following immunotherapy, however, GrzB concentrations surged to 2.0–2.5 pM (^***^ *p*< 0.001), indicating a robust restoration of T‐cell cytotoxicity. A heatmap of matched patient cohorts (Figure 5d) clearly illustrates this longitudinal upregulation, confirming that the sensor can accurately track personalized therapeutic responses.

**FIGURE 5:**
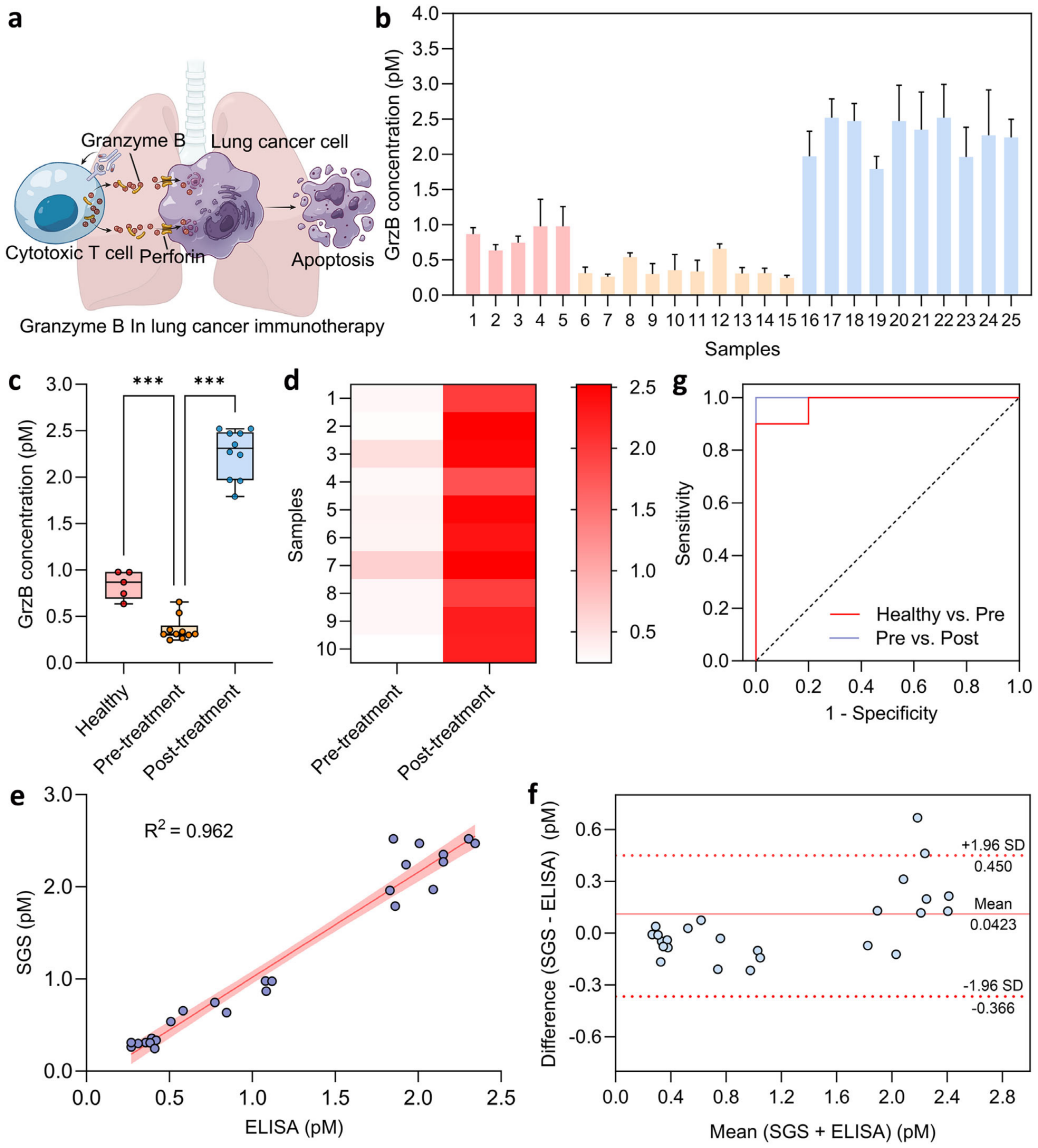
Clinical application of the proposed soft gating sensor (SGS) for monitoring lung cancer immunotherapy. (a) Schematic of GrzB release by cytotoxic T lymphocytes targeting lung cancer cells during immune checkpoint blockade therapy. (b) GrzB concentrations measured by the SGS across three groups: healthy donors (pink, *n* = 5), lung cancer patients before immunotherapy (pre‐treatment, orange, *n* = 10), and patients responding to immunotherapy (post‐treatment, blue, *n* = 10). (c) Statistical distribution of GrzB levels among the corresponding cohorts (^***^ *p*< 0.001). (d) Heatmap visualizing the longitudinal elevation of serum GrzB levels in 10 matched lung cancer patients before and after immunotherapy. (e,f) Methodological validation against a gold‐standard commercial ELISA, demonstrating high linear correlation (e) and excellent analytical agreement via a Bland‐Altman plot (f). (g) Receiver operating characteristic (ROC) curves evaluating the robust diagnostic performance of the SGS for distinguishing between healthy, pre‐treatment, and post‐treatment cohorts. All measurements were performed in triplicate. Data in (b) are presented as mean ± SD.

To rigorously validate these findings, the SGS performance was benchmarked against a commercial ELISA gold standard (Figures S32 and S33). Linear regression of the paired clinical outputs yielded a high correlation coefficient (*R* ^2^ = 0.962, Figure 5e). Furthermore, Bland‐Altman analysis (Figure 5f) demonstrated highly reliable analytical agreement with a negligible mean bias of 0.0423 pM. This high concordance confirmed that the hydrogel‐gated nanochannel effectively resisted biofouling and non‐specific binding in complex biological fluids. Supported by this validated accuracy, we evaluated the diagnostic capability of the platform via receiver operating characteristic (ROC) curves (Figure 5g). The SGS accurately discriminated between healthy donors and pre‐treatment patients, achieving an area under the curve (AUC) of 0.980, and exhibited excellent sensitivity and specificity (AUC ∼ 1.0) in categorizing positive therapeutic responses (pre‐ vs. post‐treatment). Crucially, these diagnostic metrics perfectly mirror the performance derived from the gold‐standard ELISA measurements (Figure S34), confirming that the SGS achieves equivalent diagnostic accuracy. Together, these robust metrics establish the developed nanofluidic platform as a highly reliable and practical tool for the rapid clinical monitoring of immunotherapy biomarkers. This high diagnostic accuracy in unpurified physiological fluids serves as a direct clinical validation of the 3D charge‐wettability gating mechanism. It confirms that the volumetric architecture not only resists biofouling, but successfully mitigates the Debye screening limitations that traditionally hinder clinical immunodiagnostics.

## 3 Conclusion

In summary, this work presents a 3D volumetric soft gating strategy for solid‐state nanochannel sensing. Rather than relying on traditional 2D interfacial modifications, we utilized the target‐triggered macroscopic phase disassembly of a peptide‐DNA hydrogel to create a distinct charge‐wettability synergy. This volumetric regulation mechanism, driven by macroscopic Donnan enrichment, effectively circumvented classical Debye screening limitations, significantly amplifying transmembrane ionic flux and achieving femtomolar sensitivity. Furthermore, the modularity of the supramolecular network allowed for the integration of multiplexed biochemical recognition events into a cascaded INHIBIT‐OR logic gate, enabling programmable biocomputing operations. Crucially, the intact 3D network functions as an intrinsic physical shield, ensuring anti‐fouling robustness and allowing for the direct analysis of unpurified clinical specimens. In a clinical proof‐of‐concept study, the SGS successfully tracked the longitudinal upregulation of serum Granzyme B in lung cancer patients responding to immune checkpoint blockade therapy. The platform demonstrated high diagnostic accuracy and strong analytical agreement with gold‐standard ELISA, validating its clinical stratification power for immunotherapy monitoring.

However, several engineering and translational challenges remain before this technology can be broadly employed at the point of care. At the fundamental material level, to further optimize the sensor, future designs can maximize the primary charge effect by augmenting the spatial packing density of the DNA network. Simultaneously, the secondary wettability effect can be enhanced by incorporating hydrophobic moieties into the crosslinkers, generating a sharper hydrophilic‐to‐hydrophobic transition to maximize the ultimate signal‐to‐noise ratio. Beyond molecular optimization, translating this laboratory‐based electrochemical setup into an automated, multiplexed microfluidic cassette will be necessary to reduce manual intervention and standardize batch‐to‐batch performance. In addition, given the heterogeneity of the tumor microenvironment, the current biocomputing logic gates will need to be expanded to process a wider panel of predictive biomarkers simultaneously. Future development should focus on integrating these soft‐gating interfaces into miniaturized diagnostic arrays. Addressing these technical barriers will determine whether this 3D volumetric regulation strategy can transition from a proof‐of‐concept analytical method into a viable clinical tool for precision oncology.

## Author Contributions

**Liu Shi**: Conceptualization, data curation, formal analysis, funding acquisition and writing – original draft. **Zimeng Zhang**: Data curation, investigation. **Bingheng Li**: Data curation, formal analysis. **Yalei Gao**: Data curation, investigation. **Ruirui Zhang**: Data curation, formal analysis. **Zheying Mu**: Data curation, Methodology. **Jian Ni**: writing – review and editing. **Bing Bo**: Methodology, writing – review and editing. **Genxi Li**: Conceptualization, project administration, and writing – review and editing.

## Conflicts of Interest

The authors declare no conflicts of interest.

## Supporting information




**Supporting File**: advs78049‐sup‐0001‐SuppMat.docx.

## Data Availability

The data that support the findings of this study are available in the Supporting Information of this article.
