# Supplementary material for: Hydrogel Phase Transition‐Driven Soft Gating Circumvents Debye Screening for Advanced Biosensing
Source: Adv Sci (Weinh). 2026 Sep 28:e78049. Online ahead of print. doi: 10.1002/advs.78049 (PMC13618770; doi:10.1002/advs.78049)
Supplement: Supplementary file 1 [file ADVS-9999-e78049-s001.docx]

**Supporting Information**

Hydrogel Phase Transition-Driven Soft Gating Circumvents Debye Screening for Advanced Biosensing

Liu Shi^1,*^, Zimeng Zhang^1^, Bingheng Li^1^, Yalei Gao^1^, Ruirui Zhang^1^, Zheying Mu^2^, Jian Ni^3^, Bing Bo^3,*^, Genxi Li^2,4,*^

^1^School of Environmental and Biological Engineering, Nanjing University of Science and Technology, Nanjing 210094, P. R. China

^2^State Key Laboratory of Analytical Chemistry for Life Science, School of Life Sciences, Nanjing University, Nanjing 210023, P. R. China

^3^Department of Medical Oncology, Shanghai Pulmonary Hospital & Thoracic Cancer Institute, Tongji University School of Medicine, Shanghai 200433, P. R. China

^4^Center for Molecular Recognition and Biosensing, School of Life Sciences, Shanghai University, Shanghai 200444, P. R. China

**Correspondence:** Liu Shi (liushi@njust.edu.cn) **|** Bing Bo (bbtice@163.com) **|** Genxi Li (genxili@nju.edu.cn)

Table of Contents

[1. Experimental Section	S5](#_Toc238762145)

[1.1 Materials	S5](#_Toc238762146)

[1.2 Synthesis of Peptide-DNA Conjugates (PDCs)	S5](#_Toc238762147)

[1.3 Asymmetric Functionalization	S6](#_Toc238762148)

[1.4 DNA-Polyacrylamide Conjugate Synthesis	S6](#_Toc238762149)

[1.5 Hydrogel Assembly on the Outer Surface of Nanochannels	S7](#_Toc238762150)

[1.6 Characterization and Electrochemical Measurements	S7](#_Toc238762151)

[1.7 Optimization of Experimental Conditions	S8](#_Toc238762152)

[1.7.1 Optimization of Nanochannel Size	S8](#_Toc238762153)

[1.7.2 Optimization of Linker DNA Immobilization Concentration	S9](#_Toc238762154)

[1.7.3 Optimization of Hydrogel Network	S9](#_Toc238762155)

[1.7.4 Optimization of Enzymatic Reaction Conditions	S9](#_Toc238762156)

[1.8 Calculation of Surface Charge Density	S10](#_Toc238762157)

[1.9 Finite-Element Simulations	S10](#_Toc238762158)

[1.10 Biosensing and Logic Gate Operation	S12](#_Toc238762159)

[1.11 Clinical Sample Analysis	S12](#_Toc238762160)

[1.12 Statistical Analysis	S13](#_Toc238762161)

[2. Supporting Results and Discussion	S14](#_Toc238762162)

[2.1 Systematic Optimization of Experimental Conditions	S14](#_Toc238762163)

[2.1.1 Optimization of Nanochannel Aperture	S14](#_Toc238762164)

[2.1.2 Optimization of Linker DNA Concentration	S14](#_Toc238762165)

[2.1.3 Optimization of Hydrogel Functional Zone	S15](#_Toc238762166)

[2.1.4 Optimization of Assembly Kinetics	S16](#_Toc238762167)

[2.1.5 Optimization of Enzymatic Cleavage Conditions	S16](#_Toc238762168)

[3. Supporting Tables	S18](#_Toc238762169)

[Table S1. Oligonucleotide sequences used in this study.	S18](#_Toc238762170)

[Table S2. Peptide sequences used in this study.	S18](#_Toc238762171)

[Table S3. Comparison of the developed method with other methods.	S18](#_Toc238762172)

[4. Supporting Figures	S20](#_Toc238762173)

[Figure S1. Schematic illustration of the step-by-step molecular assembly of the peptide-DNA building blocks on the nanochannel surface.	S20](#_Toc238762174)

[Figure S2. Electrospray ionization mass spectrometry (ESI-MS) characterization of the synthesized functional peptide 1.	S21](#_Toc238762175)

[Figure S3. Schematic illustration of the synthesis route for the Peptide-DNA Complex (PDC) cross-linker.	S22](#_Toc238762176)

[Figure S4. Morphological characterization of the asymmetric anodic aluminum oxide (AAO) membrane.	S23](#_Toc238762177)

[Figure S5. Schematic illustration of the stepwise chemical functionalization of the AAO membrane surface.	S24](#_Toc238762178)

[Figure S6. XPS Si 2p spectra.	S25](#_Toc238762179)

[Figure S7. XPS S 2p spectra.	S26](#_Toc238762180)

[Figure S8. Macroscopic verification of the hydrogel phase transition.	S27](#_Toc238762181)

[Figure S9. High-magnification cross-sectional SEM image of the hydrogel interface.	S28](#_Toc238762182)

[Figure S10. Water contact angle profiles tracking the phase transition.	S29](#_Toc238762183)

[Figure S11. Schematic illustration of the electrochemical measurement setup.	S30](#_Toc238762184)

Figure S12. Optimization of the nanochannel size…………………………….S31

[Figure S13. Optimization of the linker DNA immobilization concentration.	S32](#_Toc238762185)

[Figure S14. Optimization of the hydrogel network density.	S33](#_Toc238762186)

[Figure S15. Optimization of the molar ratio among the hydrogel building blocks.	S34](#_Toc238762187)

[Figure S16. Optimization of the hydrogel assembly temperature.	S35](#_Toc238762188)

[Figure S17. Optimization of the hydrogel cross-linking time.	S36](#_Toc238762189)

[Figure S18. Optimization of the buffer pH for the enzymatic cleavage.	S37](#_Toc238762190)

[Figure S19. Optimization of the incubation temperature for enzymatic cleavage.	S38](#_Toc238762191)

[Figure S20. Monitoring of the GrzB-triggered hydrogel disassembly over time.	S39](#_Toc238762192)

[Figure S21. ESI-MS characterization of the synthesized functional peptide 2.	S40](#_Toc238762193)

[Figure S22. Water contact angle measurements for the 2D control experiment.	S41](#_Toc238762194)

[Figure S23. 2D contour map for GrzB detection using the 2D control device.	S42](#_Toc238762195)

[Figure S24. Comparison of the limit of detection (LOD) achieved in this work with other reported GrzB sensing platforms.	S43](#_Toc238762196)

[Figure S25. Electrical performance of the traditional 2D surface-modified nanochannels under varying background ionic strengths.	S44](#_Toc238762197)

[Figure S26. Electrical performance of the 3D peptide-DNA hydrogel-gated nanochannels under varying background ionic strengths.	S45](#_Toc238762198)

[Figure S27. Quantitative analytical performance of the 3D soft gating sensor evaluated exclusively at physiological ionic strength.	S46](#_Toc238762199)

[Figure S28. Chemical structures of the target-specific Peptide-DNA Complexes (PDCs).	S47](#_Toc238762200)

[Figure S29. ESI-MS characterization of the synthesized functional peptide 3.	S48](#_Toc238762201)

[Figure S30. Reversibility of surface wettability during the sensor regeneration process.	S49](#_Toc238762202)

[Figure S31. Wettability evaluation during sensor regeneration.	S50](#_Toc238762203)

[Figure S32. ELISA standard curve.	S51](#_Toc238762204)

[Figure S33. ELISA quantification of clinical samples.	S52](#_Toc238762205)

[Figure S34. ELISA diagnostic performance.	S53](#_Toc238762206)

[5. Supporting References	S54](#_Toc238762207)

# 1. Experimental Section

## 1.1 Materials

DNA oligonucleotides (sequences listed in Table S1; >95% purity by HPLC/PAGE) were obtained from Sangon Biotech (Shanghai, China). Functional peptides (sequences listed in Table S2; >95% purity by HPLC) were synthesized by BankPeptide Biological Technology Co., Ltd (Anhui, China). Recombinant human Granzyme B (GrzB) and MMP-2 were purchased from Abcam and Sino Biological, Inc., respectively. 4-aminophenylmercuric acetate (APMA) was obtained from Genmed Scientifics, Inc. (USA). Anodic aluminum oxide (AAO) membranes were supplied by Hefei Puyuan Nanotechnology Co., Ltd. (3-aminopropyl)triethoxysilane (APTES), acrylamide, N,N,N',N'-Tetramethylethylenediamine (TEMED), ammonium persulfate (APS), glutaraldehyde (GA), thrombin (Thr), hemoglobin (Hb), immunoglobulin G (IgG), bovine serum albumin (BSA), and calcium chloride (CaCl₂) were purchased from Sigma-Aldrich (Shanghai, China). Potassium chloride (KCl) was obtained from Nanjing Chemical Reagent CO., Ltd (Nanjing, China). All other reagents were of analytical grade. Ultrapure water (18.2 MΩ·cm, Milli-Q) was used for all solution preparations.

## 1.2 Synthesis of Peptide-DNA Conjugates (PDCs)

PDCs were synthesized via a stepwise orthogonal conjugation strategy. Briefly, amine-modified DNA (NH_2_-DNA, 100 μM) was activated with Sulfo-SMCC (1 mM) in phosphate buffer (10 mM, pH 7.4) at 25 ℃ for 12 h to generate maleimide-functionalized DNA. After purification by centrifugal filtration (Amicon Ultra-3K), the product was reacted with cysteine-terminated peptides (1 mM) at 25 ℃ for 24 h via thiol-maleimide coupling. Subsequently, DBCO-modified DNA was conjugated to the peptide-DNA intermediate via strain-promoted azide-alkyne cycloaddition (SPAAC). The final PDCs were purified using 12% denaturing polyacrylamide gel electrophoresis (PAGE) and quantified via UV-vis spectroscopy.

## 1.3 Asymmetric Functionalization

To construct the external soft-gating interface, the AAO membranes were first hydroxylated, silanized with 5% (v/v) APTES for 12 h under continuous shaking at 200 rpm, and subsequently activated with 2.5% (v/v) glutaraldehyde at 4 ℃ for 4 h. To achieve asymmetric functionalization, the membrane was then mounted in a custom-built cell, exposing the small-diameter channel surface to the subsequent reaction reagents. Linker DNA was specifically immobilized on this GA-activated outer surface in a humid chamber at room temperature for 12 h. The films were then rinsed with PBS to remove unbound DNA. Finally, non-specific binding sites were passivated by incubating the films with 10 mg·mL⁻¹ BSA in PBS, followed by thorough PBS rinsing.

## 1.4 DNA-Polyacrylamide Conjugate Synthesis

DNA-polyacrylamide conjugates (P-SB and P-SC) were synthesized by copolymerizing 500 μM acrydite-modified DNA (SB and SC) with 2.4% (w/v) acrylamide (monomer solution in 10 mM PBS, pH 7.4). The solution was vacuum-degassed for 6 min at 37 ℃. Polymerization was initiated by introducing 1.5% (w/v) APS (freshly prepared, 10% w/v) and 2.0% (v/v) TEMED (freshly prepared, 5% v/v), followed by further vacuum deoxidation for 10 min at 37 ℃. The resulting conjugates were purified by ultrafiltration (Amicon Ultra-100K, 12,000 × *g*, 15 min) to remove unreacted monomers and short oligomers.

## 1.5 Hydrogel Assembly on the Outer Surface of Nanochannels

Hydrogel assembly was performed on the DNA-functionalized AAO surface. A precursor solution containing Strand A, P-SB, P-SC, and the GrzB-specific PDC linker in hybridization buffer (10 mM Tris-HCl, 100 mM NaCl, pH 8.0) was drop-cast onto the functionalized outer surface of the AAO. The system was thoroughly shaken at 65 ℃ for 5 min for thermal annealing, followed by driving the *in situ* hybridization-mediated assembly of the 3D supramolecular network. After that, the functionalized films were rinsed three times with hybridization buffer to eliminate unbound species.

## 1.6 Characterization and Electrochemical Measurements

Surface morphology was characterized using scanning electron microscopy (SEM, Hitachi SU8020) and atomic force microscopy (AFM, Bruker Dimension Icon). Surface chemical composition was analyzed by X-ray photoelectron spectroscopy (XPS, Thermo Fisher ESCALAB 250Xi) and Fourier-transform infrared (FTIR) spectroscopy (Bruker Optics). Confocal laser scanning microscopy (CLSM, Leica TCS SP8) was utilized to visualize the FAM-labeled hydrogel distribution. Surface wettability was quantified via contact angle goniometry (OCA 50, DataPhysics Instruments GmbH, Filderstadt, Germany). Zeta potential measurements were performed via an electrokinetic analyzer (SurPASS 3, Anton Paar, Austria). Ionic current measurements were conducted using a CHI 660F electrochemical workstation (Chenhua Instrument) in an H-type setup with Ag/AgCl electrodes. The transmembrane current was recorded in 10 mM PBS (10 mM phosphate buffer, pH 7.4, without additional NaCl) or 10 mM KCl via *I–V* sweeps from -1.0 V to +1.0 V. Furthermore, to evaluate the Donnan mechanism and device performance under physiological conditions, subsequent measurements were conducted across a broad ionic strength gradient (from 10 up to 200 mM).

## 1.7 Optimization of Experimental Conditions

To achieve optimal sensing performance, we systematically investigated several key parameters governing the hydrogel assembly and enzymatic cleavage. Across all optimization assays, the transmembrane ionic current was recorded at an applied potential of −1.0 V. The optimal conditions were determined by calculating the current blockade ratio, (Δ*I*/*I*₀)%. All optimization experiments were conducted at least three times.

### 1.7.1 Optimization of Nanochannel Size

Anodic aluminum oxide (AAO) membranes with a series of distinct channel diameters (40, 60, 100, 200, and 260 nm) but consistent thickness and material properties were selected. Functionalization, hydrogel assembly, and GrzB (50 nM) detection were performed under identical conditions to evaluate the effect of channel diameter on the current blockade ratio.

### 1.7.2 Optimization of Linker DNA Immobilization Concentration

GA-activated AAO membranes were incubated with linker DNA at different concentrations (1, 5, 10, 20, and 50 μM). Hydrogel assembly and GrzB (50 nM) detection were carried out under identical conditions to assess the influence of linker DNA immobilization concentration on the current blockade ratio.

### 1.7.3 Optimization of Hydrogel Network

The hydrogel network was optimized by systematically investigating four key parameters: (1) the total acrylamide concentration (1.6%, 2.0%, 2.4%, 2.8%, and 3.2% w/v), (2) the molar ratios of the components Strand A, P-SB, and P-SC (1:1:1, 1:2:1, 1:1:2, 2:1:1, 2:1:2, and 2:2:1), (3) the crosslinking temperature (4, 25, 37, 50, and 65 ℃), and (4) the final crosslinking time (1.5, 3, 6, 12, and 24 h). For each experiment, the hydrogel was assembled, and the optimal value for each parameter was determined by measuring the current blockade ratio after treatment with 50 nM GrzB.

### 1.7.4 Optimization of Enzymatic Reaction Conditions

To maximize the catalytic efficiency of GrzB and the subsequent phase disassembly of the hydrogel, the enzymatic cleavage conditions were systematically optimized. Using the optimized 3D hydrogel system, we evaluated the effects of three critical parameters on the sensing performance: buffer pH (6.4, 7.0, 7.4, 8.0, and 8.4), reaction temperature (25, 30, 37, 40, and 45 ℃), and incubation time (0, 15, 30, 60, 80, and 100 min). For each variable, the hydrogel-gated membrane was incubated with 50 nM GrzB under the specified conditions.

## 1.8 Calculation of Surface Charge Density

The surface charge density (σ) of the functionalized interface was quantified based on the experimental zeta potential (ζ) measurements using the Gouy-Chapman theory. The calculation assumes a symmetrical 1:1 electrolyte environment consistent with the experimental conditions (10 mM KCl). First, the Debye screening length (λ_D_) was determined at 298.15 K according to the equation^1^:

$$\lambda_{D}=\sqrt{\frac{\epsilon_{r}\epsilon_{0}RT}{2F^{2}C_{0}}}$$

where *ϵ_r_* and *ϵ*_0_ represent the relative permittivity of water and the vacuum permittivity, respectively; *R* is the universal gas constant; *T* is the absolute temperature; *F* is the Faraday constant; and *C*_0_ is the bulk electrolyte concentration.

Subsequently, the relationship between surface charge density and zeta potential was described by the equation:

$$\sigma=\frac{2\epsilon_{r}\epsilon_{0}RT}{F\lambda_{D}}\sinh\left( \frac{F\zeta}{2RT} \right)$$

The calculated surface charge densities, derived from the measured zeta potentials of the intact and GrzB-cleaved hydrogel states, were explicitly employed as boundary conditions for the space charge density in the finite-element simulations to investigate the ion transport mechanism.

## 1.9 Finite-Element Simulations

To theoretically elucidate the ionic transport properties and the underlying gating mechanism driven by the interfacial charge-wettability synergy, numerical simulations were performed using COMSOL Multiphysics 6.3. The computational domain was defined as a 2D axisymmetric nanochannel with asymmetric geometry defined with a total length of 200 nm, featuring a small-pore diameter of 0.5 nm and a large-channel diameter of 60 nm. The steady-state mass transport of each ionic species (*i* = *K*^+^, *Cl*^-^) in the electrolyte solution is governed by the Nernst-Planck equation and the continuity equation^2^:

$$\boldsymbol{J}_{i}=-D_{i}\nabla c_{i}-\frac{z_{i}F}{RT}D_{i}c_{i}\nabla\emptyset+uc_{i}$$

$$\nabla\cdot J_{i}=0$$

where$\boldsymbol{J}_{i}$, $D_{i}$, $c_{i}$, and $z_{i}$ are the ionic flux, diffusion coefficient, local concentration, and valence of species $i$, respectively. $F$ is the Faraday constant, $R$ is the universal gas constant, $T$ is the absolute temperature, $\emptyset$ is the local electrical potential, and $u$ is the fluid velocity vector.

The electrical potential distribution $\emptyset$ within the nanochannel, dictated by the spatial charge distribution, is described by the Poisson equation:

$$\nabla^{2}\emptyset=\frac{-\rho_{v}}{\varepsilon_{0}\varepsilon_{r}}=-\frac{F}{\varepsilon_{0}\varepsilon_{r}}\sum z_{i}c_{i}$$

where $\varepsilon_{0}$ is the permittivity of a vacuum and $\varepsilon_{r}$ is the relative dielectric constant of the electrolyte solution.

To accurately simulate the sensing process triggered by the hydrogel phase transitions, the boundary conditions at the nanochannel inner walls were specifically defined to represent two distinct states. In the initial hydrogel-covered state, the surface was assigned a high negative surface charge density of -5.26 mC/m² and defined with superhydrophilicity to mimic the intact hydrogel coating, which induces robust Donnan enrichment and high selective ionic conductance. Conversely, to simulate the target-induced degradation of the hydrogel in the triggered state, the surface charge density was sharply reduced to -0.92 mC/m², accompanied by diminished hydrophilicity. For the bulk environment in both simulation models, the electrolyte concentration was set to 10 mM KCl. A transmembrane potential bias of -1 V was applied across the ends of the nanochannel to evaluate the difference in ionic flux, thereby validating the amplified sensing performance achieved via the charge-wettability synergy.

## 1.10 Biosensing and Logic Gate Operation

For GrzB sensing, the functionalized device was incubated with varying concentrations of GrzB (1 fM – 1 nM) in a pH 7.4 buffer at 37 ℃ for 30 min. The current change ratio, defined as:

(∆*I* / *I*_0_)% = |(*I* − *I*_0_) / *I*_0_| × 100%

was used as the sensor response (where *I_0_* and *I* denote the ionic currents measured at an applied potential of −1.0 V before and after the reaction, respectively). For the construction of the INHIBIT-OR logic gate, a dual-functional hydrogel integrated with both GrzB-specific and MMP-2-specific PDCs was assembled. Logic operations were performed by introducing combinations of inputs (GrzB, MMP-2, Z-AAD-CMK, and Captopril) and recording the ionic current output.

## 1.11 Clinical Sample Analysis

Clinical samples were acquired from patients enrolled at the Shanghai Pulmonary Hospital between November 2025 and February 2026. Peripheral blood was collected from five healthy volunteers and a cohort of ten patients diagnosed with advanced (stage III–IV) non-small cell lung cancer (NSCLC). For the NSCLC cohort, blood draws were performed at two distinct time points: prior to treatment and four weeks following the initial administration of immune checkpoint inhibitors (e.g., pembrolizumab or nivolumab). The study protocol was approved by the Medical Ethics Committee of Shanghai Pulmonary Hospital (Approval No. K22-047Z) and was conducted in strict accordance with the ethical principles of the Declaration of Helsinki. Written informed consent was obtained from all participants prior to enrollment.

## 1.12 Statistical Analysis

Statistical analyses were performed using OriginPro (v. 2023) and GraphPad Prism (v. 10.1.2) software. All measurements were performed at least three times unless otherwise stated. Data are expressed as mean ± standard deviation (mean ± SD). For the analytical performance and device robustness assays (including logic gate operations and batch-to-batch reproducibility), linear regression analysis was applied to extract the calibration curves, limit of detection (LOD), and correlation coefficients (*R*²). For the clinical serum analysis, statistically significant differences between experimental groups (healthy donors, pre-treatment, and post-treatment cohorts) were evaluated using a two-tailed Student’s *t*-test (for comparing two groups) or a one-way analysis of variance (ANOVA) followed by Dunnett’s post hoc test (for multiple comparisons). A *P* value of < 0.05 was considered statistically significant.

# 2. Supporting Results and Discussion

## 2.1 Systematic Optimization of Experimental Conditions

To maximize the signal gating ratio of the 3D phase-transition interface and achieve ultrasensitive biosensing, we systematically optimized the structural parameters, network composition, and reaction kinetics of the hydrogel-gated device. The current blockade ratio after 50 nM GrzB cleavage was utilized as the primary evaluation metric.

### 2.1.1 Optimization of Nanochannel Aperture

The intrinsic dimensions of the solid-state nanochannels may influence the baseline ionic flux and the effectiveness of the external soft gating. We evaluated AAO membranes with diverse channel diameters ranging from 40 nm to 260 nm (Figure S12). The results indicated that the signal ratio peaked at a diameter of 60 nm. Channels smaller than 60 nm (e.g., 40 nm) suffered from excessive spatial confinement, leading to high initial resistance and limited signal modulation space. Conversely, when the channel diameter exceeded 60 nm, the massive bulk solution volume passing through the wide channel orifice dominated the ion transport. This effectively diluted the volumetric charge-wettability synergistic regulation exerted by the external hydrogel, leading to a significant decay in gating efficiency. Thus, 60 nm was selected as the optimal diameter.

### 2.1.2 Optimization of Linker DNA Concentration

The density of the anchoring sites directly determines the compactness and stability of the assembled 3D supramolecular network. We evaluated the linker DNA concentration from 1 μM to 50 μM (Figure S13). The current blockade ratio increased significantly as the concentration rose to 10 μM and plateaued at 20 μM. This indicated the formation of a robust, densely packed hydrogel that acts as a highly efficient volumetric ion trap, maximizing the baseline ionic flux (State ON) and thereby generating the largest current drop upon target-induced disassembly. However, when the concentration was further increased to 50 μM, a distinct decline in the signal ratio was observed. This reduction at excessively high concentrations was attributed to severe steric hindrance at the nanochannel entrance, which not only restricted the subsequent hybridization of the hydrogel building blocks but also hindered the accessibility of the GrzB enzyme. Consequently, 10 μM was chosen as the optimal linker concentration, as it successfully achieved maximum gating efficiency while avoiding the detrimental effects of overcrowding.

### 2.1.3 Optimization of Hydrogel Functional Zone

The physicochemical collapse of the hydrogel is dependent on its internal cross-linking density. We first modulated the total acrylamide concentration (Figure S14). The maximum gating ratio was achieved at 2.4% (w/v). Lower concentrations failed to form a sufficiently dense charge barrier, while higher concentrations yielded excessively rigid networks that resisted enzymatic disassembly. Furthermore, we investigated the stoichiometry of the building blocks (Strand A, P-SB, P-SC) (Figure S15). Both the equimolar ratio (1:1:1) and the P-SB-excess ratio (1:2:1) exhibited excellent and comparable gating performance. While the 1:2:1 configuration yielded a marginally higher current blockade ratio by further minimizing unsaturated free ends, the 1:1:1 ratio provided a highly robust synergistic shutdown effect that fully meets the requirements for ultrasensitive detection. Consequently, to strike an optimal balance between superior analytical performance and the cost-efficiency of reagent consumption for future scalable applications, the 1:1:1 molar ratio was selected as the optimal condition.

### 2.1.4 Optimization of Assembly Kinetics

The *in situ* growth of the hydrogel requires precise thermodynamic control. The assembly temperature and time were optimized to ensure comprehensive DNA hybridization and robust supramolecular cross-linking. As shown in Figure S16, 37 ℃ emerged as the optimal temperature, optimally balancing the hybridization thermodynamics of the specific DNA sequences without causing non-specific aggregation or thermal denaturation. Kinetic tracking (Figure S17) revealed that although the current blockade ratio fully plateaued after 12 hours of incubation, the supramolecular network assembled within 6 hours already exhibited a robust signal gating response. Therefore, to strike an optimal balance between operational time efficiency and robust sensing performance, a 6-hour assembly period at 37 ℃ was set as the standard protocol for all subsequent experiments.

### 2.1.5 Optimization of Enzymatic Cleavage Conditions

The cleavage efficiency of GrzB strictly governs the sensitivity of our platform. We systematically mapped the catalytic microenvironment, including pH, temperature, and reaction time. The maximum current blockade ratio was recorded at pH 7.4 (Figure S18) and 37 ℃ (Figure S19), which closely aligns with the physiological conditions favored by human GrzB. Finally, time-dependent monitoring of the ionic current (Figure S20) demonstrated a rapid and significant signal change within the first 30 minutes of adding GrzB, while the signal of the control group without GrzB remained stable. Although the signal began to gradually plateau after this initial phase, the 30-minute mark provided an ample response margin. Therefore, to balance high detection efficiency with sufficient signal amplitude, a 30-minute incubation period at 37 ℃ in a pH 7.4 buffer was established as the standard protocol for all subsequent biosensing operations.

# 3. Supporting Tables

Table S1. Oligonucleotide sequences used in this study.

| Name | Sequence (5’ to 3’) |
| --- | --- |
| Linker | NH_2_-C_6_-CAGTGTCTGTGAG |
| Strand A | CTCCAGGCAGCTCGAACTCACAGACACTG |
| Strand B | Acrydite-AAAACGAGCTGCCTGGAGAACTGCTGTCACGAG |
| Strand C | Acrydite-AAAACATGCGTATTCAT |
| NH_2_-DNA | NH_2_-TTTCTCGTGACAGCAG |
| DNA-DBCO | DBCO-ATGAATACGCATGT |

Table S2. Peptide sequences used in this study.

| Name | Sequence |
| --- | --- |
| Peptide 1 | CGGIEFDSGG-Lys(N₃) |
| Peptide 2 | CGGIEFDSGGKK-Lys(N₃) |
| Peptide 3 | CGGPLGVRSGGD-Lys(N₃) |

## Table S3. Comparison of the developed method with other methods.

| Methods | Linear range | Detection limit | Reference |
| --- | --- | --- | --- |
| Fluorescence | 10 – 300 nM | 41.6 nM | [3] |
| Fluorescence | 0 – 9.375 nM | 118 pM | [4] |
| Fluorescence | 15 – 150 pM | 15.3 pM | [5] |
| Fluorescence | —— | 1.7 pM | [6] |
| Electrochemistry | 0.094 – 1.56 pM &1.56 – 31.25 pM | 54.7 fM | [7] |
| Electrochemistry | 0.01 – 4 pM | 10 fM | [8] |
| Electrochemistry | 0.01 pM – 8 pM | 3.5 fM | [9] |
| Electrochemistry | 0.001 – 1000 pM | 0.830 fM | This work |

# 4. Supporting Figures

## **Figure S1**. Schematic illustration of the step-by-step molecular assembly of the peptide-DNA building blocks on the nanochannel surface.

Figure S2. Electrospray ionization mass spectrometry (ESI-MS) characterization of the synthesized functional peptide 1. The spectrum confirms the successful synthesis and high purity of the peptide sequence.

Figure S3. Schematic illustration of the synthesis route for the Peptide-DNA Complex (PDC) cross-linker. The synthesis relies on a stepwise orthogonal strategy involving the maleimide-thiol coupling between maleimide-activated DNA and the functional peptide, followed by strain-promoted alkyne–azide cycloaddition (SPAAC) with DBCO-DNA.

Figure S4. Morphological characterization of the asymmetric anodic aluminum oxide (AAO) membrane. SEM images showing the (a) large-diameter channel surface, (b) small-diameter channel surface, and (c, d) cross-sectional views. The red dashed box and arrow in (c) highlight the presence of the barrier layer. Note that the overall membrane thickness is approximately 60 μm.

Figure S5. Schematic illustration of the stepwise chemical functionalization of the AAO membrane surface. The process involves initial hydroxylation, aminosilanization with APTES, glutaraldehyde (GA) activation, and the final immobilization of the amino-modified linker DNA to anchor the hydrogel network.

Figure S6. XPS Si 2p spectra. Comparison of the surface composition before and after APTES Modification.

Figure S7. XPS S 2p spectra. Comparison of the surface composition before (DNA Modification) and after (Hydrogel Assembly) introduction of the sulfur-containing components, corroborating the successful functionalization.

Figure S8. Macroscopic verification of the hydrogel phase transition. Photographs demonstrating the macroscopic state of the peptide-DNA hydrogel before (left) and after (right) incubation with Granzyme B (GrzB). The intact hydrogel maintains its 3D network against gravity, whereas enzymatic cleavage results in hydrogel disassembly and flow.

Figure S9. High-magnification cross-sectional SEM image of the hydrogel interface. The image reveals the thick, 3D porous supramolecular hydrogel network successfully assembled and strongly adhering to the AAO nanochannel surface.

Figure S10. Water contact angle profiles tracking the phase transition. Photographs of water droplets on the AAO membrane surface following (a) APTES modification, (b) hydrogel assembly (superhydrophilic state), and (c) GrzB enzymatic cleavage (hydrophilic state), corresponding to the wettability data presented in Figure 2.

Figure S11. **Schematic illustration of the electrochemical measurement setup.** A two-compartment H-type cell was utilized to conduct transmembrane ionic current measurements, with the functionalized AAO membrane mounted in the center and two Ag/AgCl electrodes applying the transmembrane potential.

Figure S12. Optimization of the nanochannel size. (a–e) Current**–**voltage (*I–V*) curves before and after GrzB enzymatic cleavage on AAO membranes with different channel diameters (40, 60, 100, 200, and 260 nm). The insets show the corresponding SEM images of the pore structures. (f) Quantitative comparison of the current responses and change ratios.

Figure S13. Optimization of the linker DNA immobilization concentration. The current ratio was evaluated using varying concentrations of linker DNA (1 to 50 μM).

Figure S14. Optimization of the hydrogel network density. The effect of total acrylamide concentration (1.6% to 3.2% w/v) on the sensing performance.

Figure S15. Optimization of the molar ratio among the hydrogel building blocks. The current change ratio was evaluated under different molar ratios of Strand A, P-SB, and P-SC (ranging from 1:1:1 to 2:2:1) to identify the optimal supramolecular network configuration.

Figure S16. Optimization of the hydrogel assembly temperature. The cross-linking temperature was optimized from 4 ℃ to 65 ℃.

Figure S17. Optimization of the hydrogel cross-linking time. The current response was monitored for varying hydrogel assembly times (1.5 to 24 h).

Figure S18. Optimization of the buffer pH for the enzymatic cleavage. The GrzB cleavage efficiency was evaluated across a pH range of 6.4 to 8.4.

Figure S19. Optimization of the incubation temperature for enzymatic cleavage. The current ratio measured at temperatures ranging from 25 ℃ to 45 ℃.

Figure S20. Monitoring of the GrzB-triggered hydrogel disassembly over time. Time-dependent ionic current response of the hydrogel-gated device in the presence (GrzB +) and absence (GrzB -) of the target enzyme.

Figure S21. ESI-MS characterization of the synthesized functional peptide 2.

Figure S22. Water contact angle measurements for the 2D control experiment. Contact angles of the traditional 2D peptide monolayer-modified surface (a) before and (b) after GrzB cleavage, demonstrating limited wettability variations compared to the 3D hydrogel system.

Figure S23. 2D contour map for GrzB detection using the 2D control device. Color-mapped 2D contour illustrating the evolution of the ionic current as a function of the transmembrane potential and the logarithm of GrzB concentration (lg *C*).

Figure S24. Comparison of the limit of detection (LOD) achieved in this work with other reported GrzB sensing platforms.

Figure S25. Electrical performance of the traditional 2D surface-modified nanochannels under varying background ionic strengths. (a–e) *I*–*V* curves of the 2D nanochannel system before (red) and after (blue) target cleavage, measured in background buffers with increasing ionic strengths of 10, 50, 100, 150, and 200 mM, respectively. (f) The corresponding gating ratio plotted as a function of the background ionic strength. The sharp decline in the gating ratio at ~50 mM experimentally confirms that the shrinking Debye length severely limits the electrostatic gating effect on conventional planar architectures.

Figure S26. Electrical performance of the 3D peptide-DNA hydrogel-gated nanochannels under varying background ionic strengths. (a–e) *I-V* curves of the 3D soft gating platform before (purple) and after (green) GrzB-triggered network disassembly, measured in background buffers with increasing ionic strengths of 10, 50, 100, 150, and 200 mM, respectively. (f) The corresponding gating ratio plotted as a function of the background ionic strength. In contrast to the 2D system, the 3D macroscopic matrix maintains a robust and measurable gating response even at a high physiological salt concentration (up to 200 mM), demonstrating that the volumetric Donnan enrichment effectively mitigates the classical Debye screening limitation.

Figure S27. Quantitative analytical performance of the 3D soft gating sensor evaluated exclusively at physiological ionic strength. (a) The target-responsive gating ratio across varying concentrations of Granzyme B (GrzB) in a strict 150 mM buffer. (b) The corresponding calibration plot, demonstrating a reliable linear correlation (*R*^2^ = 0.967) over a broad GrzB concentration range from 10^2^ to 10^6^ fM, validating its sensing capability in highly screened physiological environments.

Figure S28. Chemical structures of the target-specific Peptide-DNA Complexes (PDCs). Detailed molecular structures of the functional cross-linkers engineered for (a) Granzyme B (GrzB, containing the specific IEFD sequence) and (b) Matrix Metalloproteinase-2 (MMP-2, containing the specific PLGVR sequence).

Figure S29. ESI-MS characterization of the synthesized functional peptide 3.

Figure S30. Reversibility of surface wettability during the sensor regeneration process. Water contact angle profiles of the functionalized AAO membrane after hydrogel regeneration (a) and subsequent GrzB-triggered re-cleavage (b). The successful transition confirms the robust structural reversibility and reusability of the 3D soft gating interface.

Figure S31. Wettability evaluation during sensor regeneration. Water contact angle measurements monitoring the changes in surface hydrophilicity during the regeneration and re-cleavage cycles, confirming the reversibility of the sensing interface.

Figure S32. ELISA standard curve. Calibration curve for the commercial GrzB ELISA kit used as the gold-standard reference.

Figure S33. ELISA quantification of clinical samples. Bar chart of GrzB concentrations in the 25 clinical serum samples as measured by the commercial ELISA method.

Figure S34. ELISA diagnostic performance. ROC curves derived from standard ELISA measurements for comparison with the proposed sensor.

# 5. Supporting References

1. Xu, R., Kang, Y., Zhang, W., Pan, B., Zhang, X., "Two-dimensional MXene membranes with biomimetic sub-nanochannels for enhanced cation sieving," *Nature Communications* 14 (2023): 4907, https://doi.org/10.1038/s41467-023-40742-8.
2. Su, S., Zhang, Y., Peng, S., et al., "Multifunctional graphene heterogeneous nanochannel with voltage-tunable ion selectivity," *Nature Communications* 13 (2022): 4894, https://doi.org/10.1038/s41467-022-32590-9.
3. Liu, S.-J., Ma, K., Liu, L.-S., et al., "Point-of-care non-invasive enzyme-cleavable nanosensors for acute transplant rejection detection," *Biosensors and Bioelectronics* 215 (2022): 114568, https://doi.org/10.1016/j.bios.2022.114568.
4. Wu, Y., Dong, X., Yan, Y., Yuan, K., Meng, H.-M., Li, Z., "Aptamer-Functionalized Granzyme B-Responsive Nanoprobe for Immunotherapy Assessment: Real-Time Monitoring of Cytotoxic T Lymphocyte Activation Cleavage," *Analytical Chemistry* 97 (2025): 17141-17149, https://doi.org/10.1021/acs.analchem.5c03155.
5. Zheng, J., Li, X., Cheng, H., et al., "Ratiometric fluorescence biosensing of granzyme B based on biomimetic microspheres," *Sensors and Actuators B: Chemical* 432 (2025): 137492, https://doi.org10.1016/j.snb.2025.137492.
6. Mac, Q. D., Mathews, D. V., Kahla, J. A., et al., "Non-invasive early detection of acute transplant rejection via nanosensors of granzyme B activity," *Nature Biomedical Engineering* 3 (2019): 281-291, https://doi.org/10.1038/s41551-019-0358-7.
7. Saputra, H. A., Chung, J. H., Yoon, S. H., Seo, K.-D., Park, D.-S., Shim, Y.-B., "Disposable amperometric immunosensor with a dual monomers-based bioconjugate for granzyme B detection in blood and cancer progress monitoring of patients," *Biosensors and Bioelectronics* 198 (2022): 113846, https://doi.org10.1016/j.bios.2021.113846.
8. Shi, H., Gong, Y., Liang, Q., Li, J., Xiang, Y., Li, G., "Target-Initiated Great Change in Electrochemical Steric Hindrance for an Assay of Granzyme B Activity," *Analytical Chemistry* 93 (2021): 13382-13388, https://doi.org/10.1021/acs.analchem.1c03188.
9. Qu, X., Nie, B., Zeng, Y., Sun, C., Li, W., Li, G., "A peptides-based biosensor with target-triggered charge-switchable property for simple and sensitive detection of Granzyme B," *Biosensors and Bioelectronics* 242 (2023): 115748, https://doi.org10.1016/j.bios.2023.115748.
